# Cerebral Functional Manipulation of Repetitive Transcranial Magnetic Stimulation in Cognitive Impairment Patients After Stroke: An fMRI Study

**DOI:** 10.3389/fneur.2020.00977

**Published:** 2020-09-08

**Authors:** Yamei Li, Hong Luo, Qian Yu, Longlin Yin, Kuide Li, Yi Li, Jing Fu

**Affiliations:** ^1^Department of Rehabilitation Medicine, School of Medicine, Sichuan Academy of Medical Sciences and Sichuan Provincial People's Hospital, University of Electronic Science and Technology of China, Chengdu, China; ^2^Department of Radiology, School of Medicine, Sichuan Academy of Medical Sciences and Sichuan Provincial People's Hospital, University of Electronic Science and Technology of China, Chengdu, China

**Keywords:** repetitive transcranial magnetic stimulation (rTMS), functional magnetic resonance imaging (fMRI), cognition impairment, brain activity, functional connectivity (FC), stroke

## Abstract

**Objective:** Recently, the area of repetitive transcranial magnetic stimulation (rTMS) targeting neurological rehabilitation has been advanced as a potential treatment for post-stroke cognitive impairment (PSCI). However, the underlying mechanisms remains to be elusived. This study aims to figure out cerebral functional manipulation of rTMS in patients with PSCI through using the resting-state functional magnetic resonance imaging (rs-fMRI).

**Methods:** Thirty patients with PSCI were recruited and randomly allocated into two groups: the rTMS intervention group and control group. The rTMS intervention group was given 20 min of 5 Hz rTMS (or control) over left dorsolateral prefrontal cortex (DLPFC) besides routine cognitive intervention training for 3 consecutive weeks, five times per week, on weekdays. Cognition performance was assessed by the Minimum Mental State Examination (MMSE) and Montreal cognitive assessment (MoCA). Neural activity and functional connectivity (FC) changes were acquired by rs-fMRI with fractional amplitude of low-frequency fluctuation (fALFF) and seed-based correlation analysis.

**Results:** Cognition improvements were observed both in rTMS intervention group and control group (*P* < 0.01), while the rTMS group got more significant improvent than control group (*P* < 0.05). To be specified, compared with the control group, the rTMS group got higher fALFF values in these brain regions including superior temporal gyrus, inferior frontal gyrus and parahippocampal gyrus, while lower fALFF values in middle temporal gyrus, middle frontal gyrus and fusiform gyrus. In addition, the rTMS group showed increased FC between LDPFC and toprecuneus, inferior temporal gyrus, middle and inferior frontal gyrus and marginal gyrus, while decreased FC between LDPFC and middle temporal gyrus and thalamus.

**Conclusion:** The increase and decrease of neural activity and FC in cognition-related regions detected by rs-fMRI are good indicators to clarify the underlining mechanisms of rTMS on PSCI.

## Introduction

Post-stroke cognitive impairment (PSCI) is a common complication after stroke troubling up to 75% of the survivors (1). Only half of the patients can achieve various degree of cognition recovery, while the others will still suffer cognitive impairment or even deteriorate to vascular dementia (2). PSCI inhibits the process to restore physical rehabilitations after stoke due to memory problems and poor judgment (3). Moreover, persistent cognitive deficit will result in the worse long-term outcomes such in the activities of daily living (ADL), community reintegration, and quality of life (QOL) even the physical functions (4–6). Therefore, early and effective treatment for PSCI has become one of the priorities of modern neurological rehabilitation.

Nowadays, the therapeutic strategies for PSCI are multitudinous ranging from pharmacological to non-pharmacological treatments, including some ongoing methods of computer-assisted cognitive training, physical exercise, and brain stimulations such as transcranial direct current stimulation (tDCS) and repetitive transcranial magnetic stimulation (rTMS). However, further studies need to be performed to confirm the validity and investigate the mechanisms of these stategies (7, 8). As a novel neuro-manipulated technique, rTMS has been widely used across a range of altered states including neurological and psychiatric conditions, especially popular in treating Alzheimer's disease (AD), mild cognitive impairment (MCI), depression, mental disease, and stroke with physical disorder, aphasia or dysphagia (9–15). The advantages of rTMS are non-invasively and painlessly modulating the cortical excitability (excite or inhibit) of both the stimulated region and some distant regions in the brain (16), and reorganizing functional connectivity among certain regions to ameliorate brain networks (17). However, to date, there are still limited evidences on the application of rTMS for PSCI with positive results (8, 18, 19) and very few studies investigating the underpinnings.

The changes come from rTMS in brain activity and connectivity can been detected through a variety of techniques such as transcranial magnetic stimulation (TMS), positron emission tomography (PET), near infrared spectroscopy (NIRS) (20), electro- and magnetoencephalography (EEG/MEG) (21), low-resolution electromagnetic tomography (eLORETA) (22), and finally functional magnetic resonance imaging (fMRI) (23). fMRI is rapidly becoming the most popular technique for its value of identifying the abnormalities of cortical activity and connectivity across almost every major neurological and psychiatric disease. Moreover, it promotes the detection of rTMS on neuro-manipulated mechanisms in these diseases (20). For instance, there have been a large body of studies reporting rTMS-induced beneficial effects on Alzheimer's disease, schizophrenia, and hemiplegia or aphasia after stroke, as well as investigating the neurophysiological underpinnings of these effects via the tool of fMRI (24–27). Despite its broadly use, a comprehensive understanding of the neurophysiological underpinnings of rTMS on the PSCI patients detected by fMRI is rarely reported.

Therefore, based on previous evidences that beneficial effects of rTMS on cognitive function recovery and the capability of fMRI to approach brain functional changes, we aimed to investigate the cognitive improvement of rTMS on stroke patients measured by fMRI.

So, in our study, 5 Hz rTMS (or control) were applied on the left dorsolateral prefrontal cortex (LDPFC) of stroke patients with cognition deficits. Resting state fMRI (rs-fMRI) was employed to investigate the neurophysiological evaluations, aiming to figure out the reorganization of relative cerebral function.

## Materials and Methods

### Study Design

This is a prospective, single-center, randomized, double-blind, sham-controlled trial. It was approved by the ethics committee of Sichuan Academy of Medical Sciences & Sichuan Provincial People's Hospital, in Chengdu City, Sichuan Province, the People's Republic of China, and carried out at the inpatient department of Rehabilitation in this hospital between March 2016 and March 2018. Diagnoses were performed by board-certified and sophisticated physicians according to the cerebral apoplexy diagnostic criteria established by the 4th National Cerebrovascular Disease Conference. All participants (or their legal guardians) signed informed consent forms.

This study lasted three consecutive weeks, including a total of 15 sessions of rTMS (or sham) daily on weekdays. Group allocation was done according to the random numbers table to decide giving active or control stimulation on the participants. The grouping result was controlled by a secretary not directly involved in the research. Participants and the staff who held the assessments were fully blinded to the allocation status.

### Participants

A total of thirty patients after hemorrhagic stroke with cognitive impairment were enrolled according to the inclusion and exclusion criteria as follows. The inclusion criteria were: (1) first-ever and hemorrhagic stroke with responsible lesions located in unilateral basal ganglia and/or corona radiate region confirmed by a brain computed tomography (CT) or magnetic resonance imaging (MRI); (2) stable vital signs, no deterioration of neurological symptoms; (3) ≤ 3 months from the accident; (4) aged between 50 and 75 years; (5) right-handed; (6) with cognitive disability: MMSE < 24 (junior high school and above)/20 (primary school)/17 (illiteracy), which are cutoff values for cognitive impairment according to different education level (28); (7) without severe aphasia, visual or hearing impairment so as to be capable of fulfilling the study protocol. The exclusion criteria were: (1) non-first stroke; (2) prior history of cognitive impairment, epilepsy, or psychotic disorder; (3) cognitive dysfunction comes from other causes (e.g., alcohol addiction or drug abuse); (4) any comorbidity of serious medical conditions that could influence the study; (5) metal or electronic device implants (e.g., cardiac pacemaker, a cochlear implant, deep brain stimulator, aneurysm clip, ventriculoperitoneal shunt, or internal fixation devices); (6) cranial vault defects; (7) any non-compliance with the study protocol.

### Study Intervention

#### Group Allocation

The participants were randomly assigned to two groups: the rTMS group and control group with fifteen patients, respectively. The involved domains of cognition impairment including memory, attention, orientation, visuoconstructional skill, executive capability and abstraction ability. Demographic and clinical variables did not significantly differ between the two groups (see Table 1). Routine cognitive training was given to both groups. rTMS was applied to the rTMS group while control manipulation was given to control group, for three consecutive weeks, five times per week (except for weekends).

**Table 1:** Demographic and Clinical characteristics.

**Variable**	**rTMS**	**control**	**t/χ^**2**^**	***p***
*N*	15	15	–	–
Age (years)	65.47 ± 3.68	64.53 ± 4.72	0.604	0.551
Gender M/F (%)	7/8	9/6	0.536	0.464
Education (years)	9.20 ± 2.31	9.07 ± 2.63	0.148	0.884
Duration (days)	22.73 ± 8.05	19.13 ± 7.95	1.233	0.228
Affected hemisphere R/L (%)	5/10	6/9	0.144	0.705
Lesion localization: basal ganglia/basal ganglia and corona radiate region (%)	8/7	10/5	0.556	0.456

*Mean± SD or frequency; t/χ^2^, statistics of two sample independent t-test or Chi-square test; p, probability for the statistical analysis. rTMS, repetitive transcranial magnetic stimulation; N, number; M, male; F, female; R, right. L, left; SD, standard deviation*.

#### Routine Cognitive Training

Cognitive training covered several domains of cognition and performed as follows: (1) Memory training—including photo recognition, picture sequence recall, video content retelling, etc.; (2) Attention training—including visual tracking and computer game training; (3) Orientation training—tell the position of indoor furniture after visiting a simply decorated room; (4) Visual and spatial perception training—including puzzles, mazes, objects identifying; (5) Judging and reasoning ability training—computer game training, such as “spot the differences”; (6) Executive capability training—including origami, hand-making, knot solving, and setting up daily activities for patients to complete independently. Each patient was given certain domain or entire domains training according to the MMSE and Montreal cognitive assessment scale (MoCA) results. The training for all patients was delivered by a specific professional therapist and the cognitive tasks remained the same every day for each patient during the study, lasting 3 continuous weeks (30 min/time, 1 time/day and 5 days/week for total of 15 times in 3 weeks).

#### rTMS Procedure

Stimulation was delivered through the Magstim Super Rapid Transcranial Magnetic Stimulator (Magstim Company Ltd., Whitland, United Kingdom) equipped with a figure-eight air-cooled coil (70 mm mean diameter). The coil was positioned over the left DLPFC (29), which was positioned by using a standard EEG cap, based on the 10–20 International System (F3 region) (30). The coil was tangents to the surface of the skull, so as to produce a magnetic field that penetrates the skull into the brain. The motor threshold (MT) for each patient was determined prior to treatment, which corresponded to the minimum intensity able to stimulate the motor cortex and elicit a visible contraction of the first dorsal interosseus muscle of the unaffected upper extremity in at least 5 out of 10 attempts (31). Patient relaxedly slept in a semisupine position and kept the head unmoving during stimulation. The active stimulation session was set at a frequency of 5 Hz and 100% of the individual MT with a total of 50 trains, 40 pulses in each train, separated by 25 s inter-train interval. For the control stimulation, all parameters were the same as for the active treatment, except that the coil was located perpendicular to the surface of the skull to mimic the treatment procedure but bring no significant magnetic field into the brain. The stimulation was conducted by a specific qualified therapist and each patient received a total of 15 daily rTMS sessions, with the same machine and at the same daytime, over the course of three consecutive weeks (except for weekends). The transcranial magnetic stimulations were well-tolerated by all subjects.

### Cognitive Assessment

All the participants were assessed for cognitive function at baseline and follow-up after 3 weeks' intervention via the MMSE and MoCA Beijing version, the widely used inspection tools for cognition status. The MMSE and MoCA scale cover multiple cognitive domains: MMSE assessed orientation, memory, delayed to recall, attention, force calculation, language and visual capacity, and the MOCA assessed naming, short-term memory, visuospatial abilities, executive function, abstraction, attention, concentration, language, and orientation (28, 32). Scores of MMSE range from 0 to 30 points, with higher scores indicating better cognitive function. For MoCA, cognitive impairment was defined as the score <26 (one point was added for subject with ≤12 years of education). The researcher performing the cognition assessments was blinded to the group allocation.

### MRI Data Acquisition

The MRI examination was performed before and after intervention by a Siemens Tim Trio 3.0 T MRI scanner (Siemens Medical Systems, Erlangen, Germany) equipped with an 8-channel phased-array head coil. rs-fMRI images were acquired via a gradient- echo- planar imaging (EPI) sequence in the following parameters: repetition time (TR) = 3,060 ms, echo time (TE) = 30 ms, flip angle = 90°, slice thickness = 3 mm, slice gap = 1 mm, matrix size = 64 × 64, field of view (FOV) = 192 × 192 mm^2^, and voxel size of 3 × 3 × 3 mm^3^ (totally 160 timing slices on axial view). Additional high-resolution T1-weighted structural images of sagittal view were obtained by a magnetization prepared rapid acquisition gradient echo imaging (MP-RAGE) sequence using the following parameters: TR = 1,900 ms, TE = 2.52 ms, flip angle = 90°, slice thickness = 1.0 mm with no slice gap, matrix size =448 × 358, field of view (FOV) = 256 × 256 mm^2^, and voxel size of 0.5 × 0.5 × 0.5 mm^3^ (totally 176 images, taking 6 min and 5 s). During the resting-state scan, participants were particularly instructed to keep their eyes closed, trying to “clear their mind” but not to fall asleep.

### MRI Data Processing

The pre-processing of rs-fMRI acquisitions was carried out using Data Processing Assistant for Resting-State fMRI (DPARSF) (Version 2.3, http://rfmri.org/DPARSF_V2_3), running on MATLAB R2014a toolbox (33). The first five time series were removed from initial magnetization instability and participants' adaption to the scan condition, then the remaining 155 EPI images were corrected for differences in slice timing and for movement within and across the volumes. The average head motion should be <1 mm in x, y, z direction, and angular rotation should be within acceptable limits (<1°). Afterwards, the functional scans were coregistered to their corresponding T1-weighted anatomical image, and then spatially normalized into the standardized Montreal Neurological Institute (MNI, Montreal, Quebec, Canada) space, further resampled to 3 × 3 × 3 mm^3^ of voxel size, and spatially smoothed using a Gaussian kernel of 6 mm full-width-at-half-maximum (FWHM). After that, the time series of each voxel was filtered (band pass 0.01–0.08 Hz) to remove the effects of very low-frequency drift and high-frequency noise. Finally, nuisance covariates including white matter and cerebrospinal fluid signal intensity were regressed out.

Fraction of amplitude of low-frequency fluctuations (fALFF) was calculated using the REST (version 1.8, http://www.restfmri.net/forum) software developed by Zou et al. (34) to measure the spontaneous neural activity. After pre-processing in DPARSF, a linear trend was removed, then the time series of each voxel were transformed into the frequency domain via Fast Fourier Transform (FFT) to get the power spectrum. Then the square roots of each frequency in the power spectrum were acquired and further the mean square root across a low-frequency range (0.01–0.08 Hz) was calculated, which was regarded as the ALFF index (35). fALFF refers to the ratio of the sum of the amplitudes at a low-frequency range (0.01–0.08 Hz) to the amplitudes of entire frequency range. At last, the acquired spatial fALFF maps were normalized with the fALFF value of each voxel divided by the whole-brain mean fALFF value and then called as “mfALFF” spatial maps.

The seed-based correlation analysis was used to detect the effect of local neural activity changes on whole brain functional connectivity (FC). The left DLPFC was defined as seed or region of interest (ROI) for FC analysis relying on the REST software. After filtering (band pass 0.01–0.08 Hz) and linear trend removed, the time series for each voxel within each seed were extracted in a sphere region (radius = 5 mm) and averaged over all voxels within the seed to acquire the mean time series of the seed region. Then Pearson's correlation analysis between the mean time series from in each seed region and that of every voxel in the whole brain was computed for a map of correlation coefficients, which were transformed to z-scores using the Fisher r-to-z transformation to improve normality and then called as z-FC maps.

### Statistical Analysis

Clinical statistical analysis was performed using the SPSS Statistics 24.0 (IBM, NY, USA). Categorical variables were presented as absolute frequencies and percentages, whereas continuous variables were presented as means ± standard deviations (SDs). Differences between categorical variables were analyzed by Chi-square test while that between continuous variables were calculated by *t*-test. *P*-values were based on two-sided tests and compared to a significance level of <0.05.

Statistical analysis of fMRI data was conducted using Statistical Parametric Mapping package (SPM8, https://www.fil.ion.ucl.ac.uk/spm/), running on MATLAB. Two groups of both fALFF and FC maps after rTMS or control manipulation treatment were compared with a two-sample *t*-test, while changes post- and pre-rTMS (or control) treatment in each group were performed with a paired *t*-test. AlphaSim correction was adopted to conduct the multiple comparisons. The probability of false-positive detection was set to *P* < 0.05, and areas with a minimum cluster size of 82 contiguous active voxels were identified as significant regions.

In the rTMS group, Pearson's correlation was performed using multiple regression in the SPM8 to assess the relationship between the MoCA score and the seed-based FC alterations, with a significance level of <0.05.

## Results

### Cognition Outcome

According to the cognitive assessment results, the details of involved cognitive domains were displayed as follows (Table 2). No differences were found between the rTMS group and the control group before intervention. There were significant improvements on cognition manifestation between pre-post scores for both groups. Moreover, the direct comparison of post differences between the two groups showed more significant improvement for the rTMS stimulation. See Table 2 for details.

**Table 2:** Details of cognition tests.

**Cognitive domains**	**rTMS group**		**Control group**	
	**Before**	**After**	**Before**	**After**
Memory (*n*)	15	13	15	12
Attention (*n*)	12	10	11	9
Orientation (*n*)	10	7	12	10
Visuoconstructional skill (*n*)	15	14	15	15
Executive ability (*n*)	15	15	15	14
Abstraction ability (*n*)	15	14	14	14
MMSE	18.67 ± 3.90^a^	23.53 ± 3.23	19.13 ± 3.48	20.60 ± 3.16
Total score				
MoCA	20.47 ± 2.80^b^	24.40 ± 2.35	20.93 ± 3.04	21.80 ± 2.76
*t*-value/*p*	0.346/0.732^a^	0.438/0.665^b^	15.128/ < 0.001^c^	15.849/ < 0.001^d^
	6.813/ < 0.001^e^	3.389/0.004^f^	2.516/0.018^g^	2.778/0.01^h^

*n is the number of patients impaired; score values are mean± SEM*.

a *Comparison of MMSE between the two groups before intervention*;

b *comparison of MoCA between the two groups before intervention*;

c *comparison between pre-post scores of MMSE for the rTMS group*;

d *comparison between pre-post scores of MoCA for the rTMS group*;

e *comparison between pre-post scores of MMSE for the control group*;

f *comparison between pre-post scores of MoCA for the control group*;

g *comparison of MMSE between the two groups after intervention*;

h *comparison of MoCA between the two groups after intervention*.

### Lesion Location

Five out of the 15 patients in the rTMS group and six out of the 15 patients in the control group suffered right hemispheric lesion. So, the activation maps of those patients were flipped along the midsagittal plane to make the images of affected hemisphere corresponded to the left side for all patients.

### rTMS Effects on fALFF

**Figure 1:**
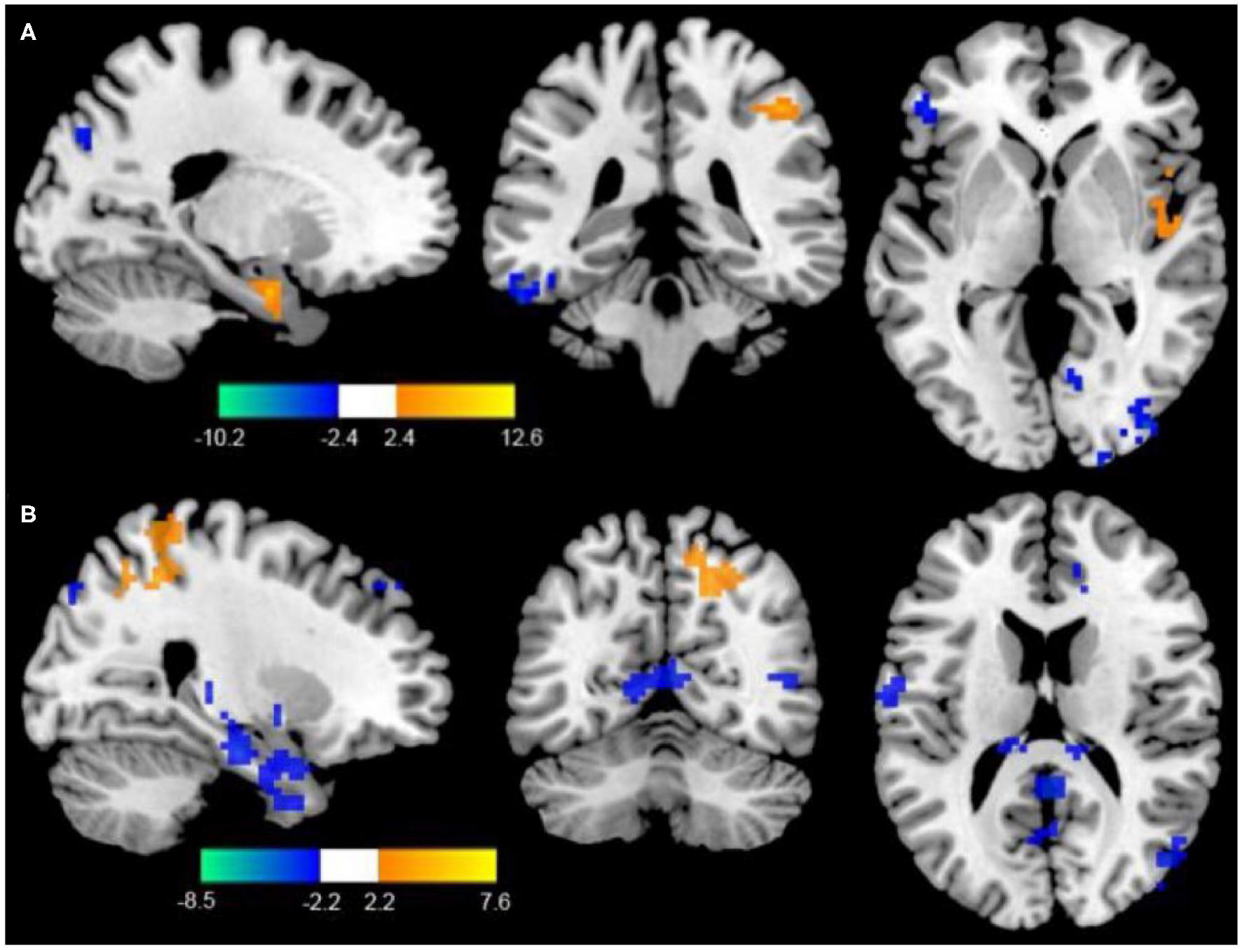
**(A)** Differences of fALFF between the rTMS treatment group and the control group (two-sample *t*-test, *P* < 0.05, AlphaSim correction, cluster size ≥ 82 voxels). The yellow areas represent the regions which have increased fALFF, while the blue ones represent the regions which have decreased fALFF. **(B)** Differences of FC with the LDPFC between the rTMS treatment group and the control group (two-sample *t*-test, *P* < 0.05, AlphaSim correction, cluster size ≥ 85 voxels). The yellow areas represent the regions which have increased FC with the LDPFC, while the blue ones represent the regions which have decreased FC with the LDPFC.

Compared to the control group, patients in rTMS group after intervention got higher fALFF values in these brain regions including the superior temporal gyrus (STG), inferior frontal gyrus (IFG), and parahippocampal gyrus, while lower fALFF values in the middle temporal gyrus (MTG), middle frontal gyrus (MFG), and fusiform gyrus (*P* < 0.05, with AlphaSim correction) (Table 3 and Figure 1A).

**Table 3:** Significant differences in regional fALFF between the two groups after rTMS or control stimulation.

**Brain region**	**Brodmann area**	**MNI coordinates**			**Clusters size**	***t*-value**
		**x**	**y**	**z**		
Regions showing increased fALFF in the rTMS group relative to the control group						
Superior temporal gyrus	22	39	24	−18	149	8.49
Inferior frontal gyrus	47	6	−57	−27	120	10.03
Parahippocampal gyrus	36	3	6	−21	138	13.78
Regions showing decreased fALFF in the rTMS group relative to the control group						
Middle temporal gyrus	21	−54	0	−27	136	−12.39
Middle frontal gyrus	11	45	36	−21	165	−6.65
Fusiform gyrus	36	−6	−27	3	139	−10.03

### rTMS Effects on Functional Connectivity

Compared with the control group, the rTMS group showed significantly increased FC between the LDPFC, toprecuneus, inferior temporal gyrus (ITG), MFG, IFG, and marginal gyrus, while decreased FC of the LDPFC was demonstrated in the MTG and thalamus (two-sample *t*-test, *P* < 0.05, AlphaSim correction, cluster size ≥ 82 voxels) (Table 4 and Figure 1B).

**Table 4:** Significant differences in FC between the two groups after rTMS or sham stimulation.

**Brain region**	**Brodmann area**	**MNI coordinates**			**Clusters size**	***t*-value**
		**x**	**y**	**z**		
Regions showing increased FC with the DLPFC in the rTMS group relative to the control group						
Precuneus	4	54	−66	12	150	7.21
Inferior temporal gyrus	20	36	3	−27	128	7.72
Middle frontal gyrus	11	18	51	30	147	4.76
Inferior frontal gyrus	47	48	0	33	180	3.79
Marginal gyrus	29	36	3	−27	134	7.29
Regions showing decreased FC with the DLPFC in the rTMS group relative to the control group						
Middle temporal gyrus	21	−60	−51	3	106	−7.31
Thalamus	26	−15	−27	12	125	−7.21

### Correlation Between fMRI and Cognition

In patients with rTMS stimulation, correlation analysis between FC values of regions with enhanced connectivity and cognitive manifestation after rTMS stimulation was calculated. The results showed that the DLPFC-precuneus/MFG/IFG/marginal gyrus FC values were positively correlated with the MoCA score (*r* = −0.839/0.776/0.842/0.796, *P* < 0.01, AlphaSim correction) (Table 5).

**Table 5:** Significant differences in correlation between FC values and MoCA sore.

**Brain region**	**Brodmann area**	**MNI coordinates**			**Clusters size**	***R***	***p***
		**x**	**y**	**z**			
Precuneus	4	54	−66	12	150	0.839	<0.001
Inferior temporal gyrus	20	36	3	−27	128	−0.785	<0.001
Middle frontal gyrus	11	18	51	30	147	0.776	<0.001
Inferior frontal gyrus	47	48	0	33	180	0.842	<0.001
Marginal gyrus	29	36	3	−27	134	0.796	<0.001

## Discussion

To the best of our knowledge, rTMS can modulate cortical excitability and may be a potential tool for cognition recovery in situations like AD, MCI, traumatic brain injury (TBI) and depression (10, 11, 36, 37), while was relatively less reported on stoke (8, 18). However, the neuroplasticity effects associated with beneficial cognitive effects from rTMS intervention still need to be thoroughly unraveled. Only few studies studied the effects of rTMS on cerebral functional reorganization in the poststroke motor dysfunction (26) or aphasia (27). Particularly, none of the previous studies have investigated the cognition recovery, as well as concerned rTMS-induced local neural activity changes and FC modulations in patients after hemorrhagic stroke. As the improvements in technology, a number of assessment methods can integrate well with rTMS to find the following neuroplasticity effects. Especially the neuroimaging tool-fMRI, which is able to provide rich information about brain activity and connectivity of various neural networks. It also could be an ideal device to reveal the underlying mechanisms of rTMS-induced neural reorganization in stroke rehabilitation (38).

To address the aforementioned limitations as mentioned above, in this study we conducted a randomized, sham-controlled clinical trial to investigate whether application of 5 Hz rTMS over the left DLPFC in stroke patients would improve cognitive manifestation, at the same time, explore whether rTMS could improve activity of cognitive related regions and modulate FC between the stimulated region with other areas in the cognition processing network. Notably, the results revealed significant cognitive improvements in patients with hemorrhagic stroke who received active rTMS intervention compared to control stimulation. Meanwhile, rs-fMRI demonstrated rTMS-induced neuroplasticity both in the stimulated regions and the other regions relate to functional network.

### The Stimulation Site and Frequency of the rTMS Treatment

The default mode network (DMN), as a main resting state networks (RSNs) in the brain, plays a important role in cognition processing. Abnormalities of DMN activity are also involved in cognition deficits (39). DLPFC is a key node in the central executive network (CEN) (40), which is closely associated with mediating executive functions and particularly linked to the activity of the DMN (41). rTMS stimulating at the DLPFC is possibly to impact the entire DMN networks, particularly the medial prefrontal cortex (mPFC) (42), which is a key hub of the DMN. Moreover, previous studies have reported that high frequency rTMS to the left DLPFC or low frequency rTMS to right DLPFC could improve cognition functions in conditions including AD, MCI, bipolar depression and stroke at different degrees (8, 10, 22, 43). However, The evidences still not enough to show the best frequency and stimulation side for rTMS on treating these diseases, although high-frequency rTMS was prone to achieve better outcomes in some conditions like Alzheimer's disease and post-traumatic stress syndrome with less adverse effects (36, 44). In this regard, the current study chose left DLPFC as the targeted site with high frequency of 5 Hz.

There's two models of reorganization for non-invasive brain stimulation (NIBS) like rTMS in stroke recovery—interhemispheric competition and vicariation. Interhemispheric competition model supports that decreasing activity of the unaffected hemisphere with low frequency stimulation would be beneficial for stroke recovery by relieving the interhemispheric inhibition for the affected hemisphere. However, the vicariation model suggests that activity in the unaffected hemisphere serves as compensation for those functions lost by affected side. The two models lead to opposite conclusions about whether the given stimulation would be inhibitory or excitatory, ultimately affecting the therapeutic effect. By introducing a new parameter “structural reserve” describing the remaining functional neural output, the different judgements are unified by integrating the two models into a new model—the bimodal balance–recovery model, which proposes that the interhemispheric competition model can predict recovery better than vicariation model in patients with high functional reserve, otherwise the vicariation model is more useful in predicting recovery (45). Therefore, studying how to measure structural reserve, including clinical, anatomical and functional reserve in future researches, plays a key role in determining optimal stimulation site and frequency for an individual patient to improve efficacy of the treatment.

### Improvements in Cognitive Functions After rTMS Treatment

In the present study, patients with cognitive impairment after hemorrhagic stroke were enrolled. Cognitive status were assessed by testing MMSE and MoCA scales. Our study showed that both the rTMS treatment and cognitive training can facilitate cognition recovery of stroke patients. Moreover, the combination of the two measures may amplify the profit of cognition enhancement. This result is also in line with several studies now available showing cognitive improvement after rTMS application for patients with stroke or Alzheimer's Disease (8, 46). Looking at the side effects of rTMS intervention, several patients experienced transient dizziness or headache in the rTMS group, and two patients complained light dizziness in the control group. These symptoms disappeared quickly without any specific interventions and no patient dropped-out of the study as to the adverse reactions. Therefore, rTMS assumed to be a safe, well-tolerated and efficacy intervention in treatment of stroke patients with cognition disorders. However, the preliminarily encouraging result needs larger controlled trials to further confirm the effectiveness of rTMS on PSCI.

### Neural Activity and FC Changes After rTMS Treatment

Intrinsic activity of the brain is organized into networks which consist of many different nodes (47). Although the brain is constrained by the anatomical skeleton, the activities of each node and functional connectivity between any two nodes within these networks are dynamic (48). By means of spontaneous low-frequency oscillations in the blood oxygenation level-dependent (BOLD) signal, the resting-state functional magnetic resonance imaging (rs-fMRI) has provided a task-free approach which could eliminate some performance-related confoundings and provide a reliable measure of “baseline” brain activity and connectivity (49). Therefore, with the help of rs-fMRI, this study focused on characterizing how the cognitive-related networks dynamically change to unravel the mechanisms of cognition recovery after rTMS treatment.

The amplitude of low frequency fluctuations (ALFF) value is a sensitive index of resting state fMRI BOLD signal (0.01–0.08 or 0.10 Hz), reflecting the amplitude of spontaneous neural activity in specific regions. While the fractional ALFF (fALFF) represents the ratio of low-frequency to the entire frequency range, which is superior to ALFF at suppressing noise components so as to enhance the sensitivity and accuracy of brain activity detection in resting state (48). Here, we found that the fALFF values of the rTMS group after intervention was significantly higher in the brain regions including the STG, IFG, and parahippocampal gyrus as compared to that of the sham group, while the values were lower in the MTG, MFG, and fusiform gyrus. The anterior STG is zoned as Wernicke region, functioning as the center of auditory information processing. The damage of Wernicke region may result in sensory aphasia which characterized by obvious auditory comprehension dysfunction and ultimately affect the cognition function for the poor words working memory. The increase of fALFF value in the STG may be related to the functional improvement of Wernicke area after rTMS treatment, accompanied by corresponding cognition improvement. The IFG is an important part of Broca region, and some studies have found that it is related to semantic acquisition and working memory (50). Increases of its fALFF value in this study again suggest that it plays an important role in cognitive function. The parahippocampal gyrus, as part of the medial temporal lobe (MTL) which is associated with memory encoding, storage, and retrieval, links the hippocampus, the retrosplenial cortex and the prefrontal cortex, plays a great part in episodic memory encoding, and retrieving (51). Therefore, the changes of the fALFF scores in these regions indicate that high-frequency rTMS over left DLPFC can facilitate corticospinal excitability, thus facilitating cognition recovery of stroke patients.

Functional connectivity (FC) analysis is extensively employed to evaluate correlations in activation among spatially-distinct brain regions for several functional neuroimaging methods, particularly fMRI either in a resting state or when processing external stimuli (38). The better correlation denotes more similarity of neural activities and stronger functional connectivity between regions (52). The rs-fMRI FC results in our study demonstrated a few discrepant brain areas between the two groups after the rTMS treatment, that is, the rTMS treatment group showed increased FC between the LDPFC and precuneus, MFG, IFG, ITG, and marginal gyrus, while decreased FC between the LDPFC and MTG and thalamus. Precuneus serves as a functional core within the default mode network. It simultaneously interacts with both the default-mode and frontoparietal networks to distinguish distinct cognitive states and plays a pivotal role in episodic and autobiographical memory (53), its latter part is also closely related to the conscious ephemeral memory (54). Frontal lobe is a highly evolved cerebral region, and richly interconnected with other cortical and subcortical structures through both short and large white matter pathways. It mainly works for integrating the afferent information from other brain regions and organizing efferent impulses timely, to ensure the overall synergy between the nervous system and psychosocial processes. It involves in a variety of higher functioning processing, including memory, abstract thinking, judgment, emotion, personality and impulsive behavior, etc (55–59). Hence, the enhancement of functional connections in frontal lobe with the LDPFC, such as MFG and IFG, has a strong connection with the improvement of cognitive function. The posterior MTG and the posterior fusiform gyrus are known to be involved in reading, and neuroanatomy has suggested that the region between the two is the posterior part of ITG which may play a core role in word cognition (60). Meanwhile, correlation analysis between FC values and cognitive manifestation after treatment in the rTMS treatment group showed that the DLPFC-precuneus/MFG/IFG/marginal gyrus FC values were positively correlated with the MoCA score. Therefore, the enhancement of functional connections in the above brain regions may be an important finding to understand the underlying mechanism of brain functional reorganization of high-frequency rTMS stimulation over the left LDPFC to facilitate cognitive function for stroke patients. As to the regions with decreased FC values, MTG plays a distinctive role in visual information processing, and its posterior part is closely connected with the language control area of the frontal parietal lobe so as to have more enhanced neural activity when performing language function (61). The thalamus is a subcortical relay station for various sensory and somatic motor signals, which plays an important role in advanced cognitive functions such as memory and emotion. After application of high frequency rTMS to the left LDPFC, the functional connections between LDPFC and the MTG and thalamus were significantly reduced, suggesting that this stimulation in our study may not have an obvious effect on improving the function of the MTG and thalamus.

### Limitations of the Study

There are a number of methodological limitations to our study. First of all, we haven't assessed the immediate after-effects of rTMS on cognition-related regions by means of fMRI, since our fMRI acquisition was done at least 24 h after intervention. Therefore, our data doesn't represent short-term rTMS-induced neuroplastic effects. Furthermore, although comparisons of patient assessments at baseline showed no statistical differences, the interindividual variability in the neural substrate such as lesion site and volume, as well as given treatments like coil positioning may confound the results from the rTMS intervention to a certain extent. Further studies will focus on individual structural or functional mapping and stereotaxy to target and stimulate the precise brain regions by combining rTMS and fMRI. Finally, for patients with different degrees of cognition defect, we might failed to control all the patients keeping “clear their mind” but not falling asleep during the relative long scanning procedure, as such, it may cause another interindividual variability and influence the evaluation accuracy of the rTMS effect. Future studies should be conducted to ensure the consistency of rest-state while fMRI acquisition.

## Conclusion

According to our study, high frequency stimulation of rTMS over left LDPFC can help to facilitate recovery of cognition in patients with hemorrhagic stroke. Our study also used the non-invasive rs-fMRI to observe the cerebral functional reorganization after the rTMS treatment and found increased or decreased neural activity and FC in some significant cognition-related regions. From this perspective, rTMS may be a crucial and safe rehabilitation tool to enhance cognition rehabilitation for stroke patients, and the rs-fMRI is good method to provide unprecedented insights into both local and functional network levels of cerebral effects stimulated by rTMS.

## Data Availability Statement

The datasets generated for this study are available on request to the corresponding author.

## Ethics Statement

The studies involving human participants were reviewed and approved by Sichuan Academy of Medical Sciences & Sichuan Provincial People's Hospital ethics committee. The patients/participants provided their written informed consent to participate in this study.

## Author Contributions

QY and YaL designed the study and performed critical revision of the article. YaL, HL, KL, and JF performed the data collection. YaL and YiL analyzed the data. YaL wrote the article. All authors contributed to the article and approved the submitted version.

## Conflict of Interest

The authors declare that the research was conducted in the absence of any commercial or financial relationships that could be construed as a potential conflict of interest.
